# The Role of DNA Methylation in Ischemic Stroke: A Systematic Review

**DOI:** 10.3389/fneur.2020.566124

**Published:** 2020-10-27

**Authors:** Minyan Zeng, Juanying Zhen, Xiaodan Zheng, Hongyan Qiu, Xiaonan Xu, Jun Wu, Zhijian Lin, Jun Hu

**Affiliations:** ^1^Department of Neurology, Peking University Shenzhen Hospital, Shenzhen, China; ^2^Department of Clinical Medicine, Shantou University Medical College, Shantou, China

**Keywords:** DNA, methylation, ischemic, stroke, systematic, review

## Abstract

**Background:** Knowledge about the classic risk and protective factors of ischemic stroke is accumulating, but the underlying pathogenesis has not yet been fully understood. As emerging evidence indicates that DNA methylation plays a role in the pathological process of cerebral ischemia, this study aims to summarize the evidence of the association between DNA methylation and ischemic stroke.

**Methods:** MEDLINE, EMBASE, PubMed, and Cochrane Central Register of Controlled Trials were searched for eligible studies. The results reported by each study were summarized narratively.

**Results:** A total of 20 studies with 7,014 individuals finally met the inclusion criteria. Three studies focused on global methylation, 11 studies on candidate-gene methylation, and six on epigenome-wide methylation analysis. Long-interspersed nuclear element 1 was found to be hypomethylated in stroke cases in two studies. Another 16 studies reported 37 genes that were differentially methylated between stroke cases and controls. Individuals with ischemic stroke were also reported to have higher acceleration in Hanuum 's epigenetic age compared to controls.

**Conclusion:** DNA methylation might be associated with ischemic stroke and play a role in several pathological pathways. It is potentially a promising biomarker for stroke prevention, diagnosis and treatment, but the current evidence is limited by sample size and cross-sectional or retrospective design. Therefore, studies on large asymptomatic populations with the prospective design are needed to validate the current evidence, explore new pathways and identify novel risk/protective loci.

## Introduction

Stroke is one of the major causes of death and disability worldwide, leading to substantial public health issues and medical costs. Currently, the global burden of stroke remains high with 80.1 million prevalent cases and 5.5 million deaths in 2016 (1). Besides, stroke burden has also been increasing in adults aged under 64 years (2, 3), suggesting that scaled-up prevention strategies with wider coverage are needed.

The knowledge of the classic factors which are associated with ischemic stroke (IS) is accumulating, mostly in the aspects of demographic characteristics, psychosocial status, cognitive function, health behavior, medication use, and cardiometabolic comorbidities (4). However, the underlying pathogenesis has not yet been fully understood. The substantial advance in the research of epigenetic modifications might provide new insights into this field and help understand additional pathological mechanisms (5, 6). DNA methylation is one of the most understood epigenetic mechanisms (7). It refers to the process of one or more methyl groups being added to a cytosine residual without changing the DNA sequence, which thereby modulates gene transcription and expression as well as many other cellular processes (8). Since DNA methylation is influenced by many environmental exposures throughout the life course (9, 10), it reflects the environment-gene interaction. It has been proved to be associated with some common diseases such as cancer (11–14), psychiatric disorders (15), and dementia (16). Emerging evidence indicated the multi-faceted role of DNA methylation in various pathological mechanisms of cerebral ischemia (6). One of the mechanisms might be its promoting effects on neuronal cell death, as researchers observed that mice who expressed lower levels of DNA methyltransferase, a catalyst of DNA methylation, were protected from cerebral ischemia (17). Other possible mechanisms include deficiency of methylenetetrahydrofolate reductase (*MTHFR*), X chromosome inactivation, aberrant homeostasis regulation, increasing oxidative stress, and abnormal modulation of synaptic plasticity (6, 18, 19). Since DNA methylation is modifiable by lifestyle factors and medical intervention, such as mental, social, and dietary factors (20, 21) it might be a promising biomarker for stroke prevention, diagnosis, and targeted neuroprotective therapy.

We aimed to conduct a systematic review according to current research literature to investigate the association of DNA methylation with the occurrence of IS. This will help update the latest evidence of the potential role of DNA methylation in cerebral ischemia and provide new insights for future research.

## Methods

### Literature Search

The systematic review was conducted in line with the Preferred Reporting Items for Systematic Review and Meta-Analyses (PRISMA) guidelines (22). We conducted the literature search systematically in four databases—MEDLINE, EMBASE, PubMed, and Cochrane Central Register of Controlled Trials from inception to April 29th, 2020. To supplement the searching result of electronic databases, we manually searched the included studies' references and unpublished studies from The Preprint Server for Biology as well as The Preprint Server for Health Science. A series of text terms and thesaurus related to DNA methylation and IS were used, with the detailed search strategies included in Supplementary Table 1. To make the search results more comprehensive, “s-adenosylmethionine,” a unique methyl donor in DNA methylation, was included in the search terms. We also included “CpG islands” as these are regions in gene promotor in which methylation is associated with epigenetic silencing (23, 24). The literature search was restricted to human studies.

### Study Selection

Two reviewers (MZ and JZ) independently screened the titles and abstracts to initially assess the relevance of studies to this systematic review. Studies meeting the inclusion criteria were subsequently assessed by full-text reading. Studies were included if they quantitatively assessed the association between the level of DNA methylation (global, candidate-gene, or genome-wide) and the diagnosis of IS. Only the studies that involved at least one or more individuals with a diagnosis of IS, or followed individuals until such a diagnosis was made were eligible. No restrictions on methods/approaches for IS diagnosis or DNA methylation measures were imposed. Studies were excluded if they: (1) were animal studies, editorials, erratum, letters, reviews, and case reports; (2) investigated irrelevant outcomes, exposures, or comparisons. A third reviewer (ZL or JH) was involved for consensus after discussion if there was a discrepancy between the results from the two reviewers in the initial screening.

### Data Extraction

Two reviewers (MZ and JZ) independently extracted the relevant data from the full-texts and supplementary materials from the eligible articles using a standardized extraction form. Data extracted included study design, characteristics of the study subjects, IS diagnostic approach, tissue sources of DNA, genes of interest, platforms of DNA methylation analysis, methylation patterns, and main findings relevant to the aim of this review. Discrepancies between the two reviewers were resolved through discussion and consultation with a third reviewer (ZL or JH).

### Assessment of Methodological Quality

The Newcastle-Ottawa Scale (NOS) was used to assess the risk of bias and quality of the studies (25). This scale is focused on several aspects, including the selection of study participants, comparability, and the measurement of exposure and outcome. The score of NOS ranges from 0 to 9 for case-control, cohort studies, and cross-sectional studies. A study with 6 stars or lower was regarded as a high risk of bias; 7 or 8 stars as medium risk of bias; 9 stars as low risk of bias. Two reviewers (MZ and JZ) independently conducted the quality assessment, and disagreements were resolved by discussion and reconfirmation with a third reviewer (ZL or JH).

### Data Synthesis

Results were summarized narratively. Data synthesis was not conducted due to the heterogeneity across studies.

## Results

### Search Results

**Figure 1:**
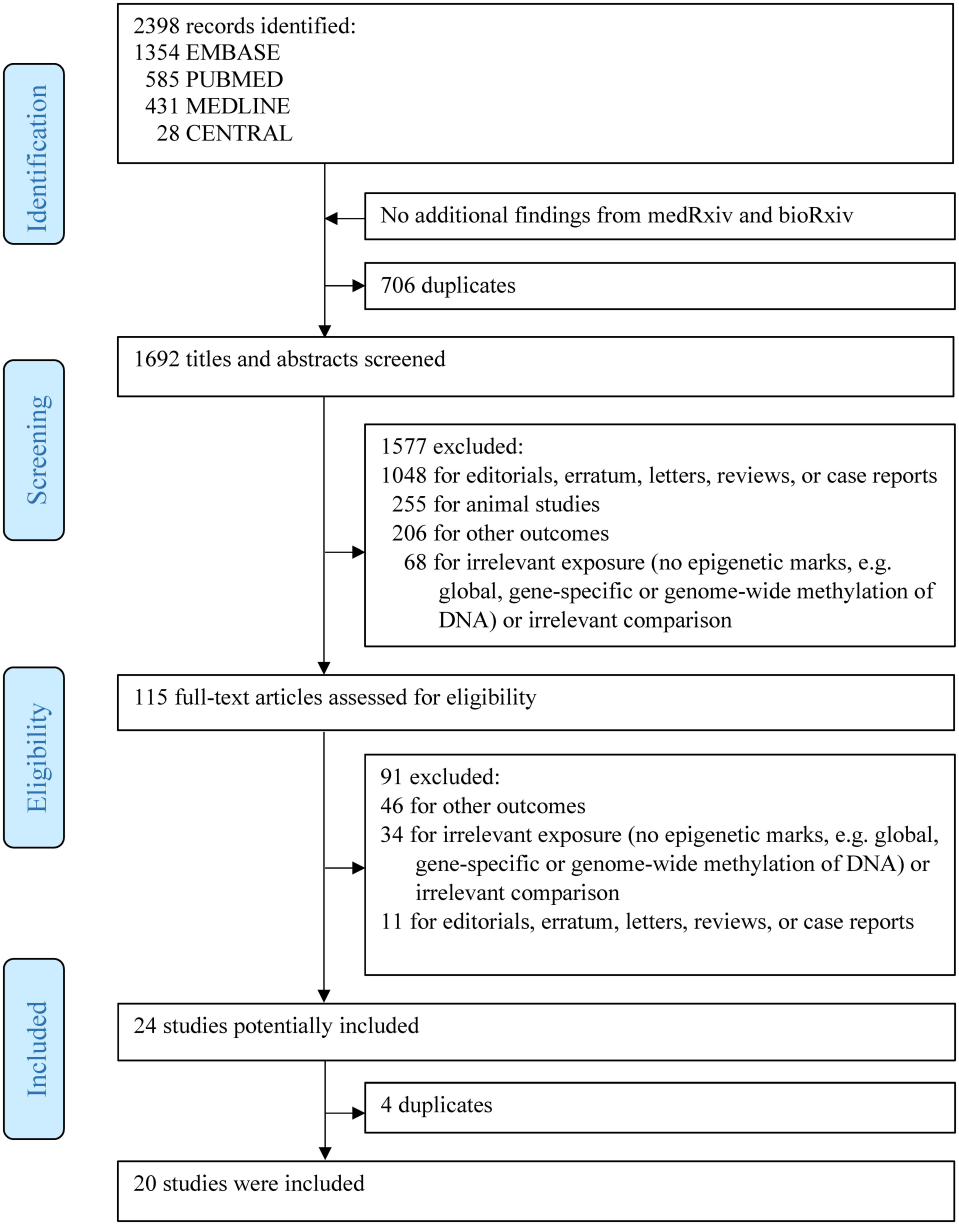
Flowchart of study selection.

As shown in Figure 1, a total of 2,398 articles were identified via the initial search. Subsequently, 1,577 articles, including editorials, erratum, letters, reviews, case reports, animal studies, and irrelevant articles, were further excluded. After full-text assessment of the remaining 115 articles, 20 articles with 7,014 individuals fulfilled the inclusion criteria and were finally included in this systematic review.

### Summary of Findings

The characteristics of eligible studies were shown in Supplementary Table 2. Of the 20 studies, 17 were case-control studies (26–42), two were prospective cohort studies (43, 44), and one was a cross-sectional study (45). Participants of the included studies were mostly older adults, with mean age ranging from 47.4 to 75.0 years. Nineteen studies recruited both male and female participants, while one study included males only (44). Eighteen studies had an independent evaluation for the diagnosis of IS and claimed the use of imaging tests such as brain computed tomography, magnetic resonance imaging, or magnetic resonance diffusion weighted imaging (26–42, 44). Nineteen out of 20 studies assessed DNA methylation from acute/subacute stroke patients (26–44). Seventeen studies were assessed as high or medium-quality, and three studies were evaluated as low-quality (Supplementary Table 3 for case-control studies, Supplementary Table 4 for cohort studies and cross-sectional studies).

Tables 1–3 summarizes the main relevant findings from the eligible studies. Fourteen studies extracted DNA from whole peripheral blood and the rest from peripheral blood leukocytes (*n* = 6). Eighteen studies reported differential patterns of DNA methylation between individuals with and without IS, but results were unable to be synthesized for a meta-analysis due to high heterogeneity across studies. Therefore, relevant results are narratively summarized below.

**Table 1:** Global methylation analysis for ischemic stroke.

**Study**	**Sample size**	**Tissue**	**Methylation sites, platform**	Methylation pattern^*^	**Main relevant findings**
(35)^a^	Case: *n* = 280 Control: *n* = 280	Peripheral blood leukocytes	*LINE-1* repetitive elements, pyrosequencing	Hypomethylation	• Mean methylation level: case vs. control, 75.9 vs. 77.0%, *p* = 0.0024; male: case vs. control, 75.7 vs. 77.3%, *p* = 0.0012; female, case vs. control, 76.2 vs. 76.7%, *p* = 0.3010. • Men: per 1% decrease in *LINE-1* methylation level, OR = 1.20, 95% CI: 1.10–1.32, *p* < 0.0001; women: not stated.
(43)	Cohort: *n* = 286 (Stroke: *n* = 4)	Peripheral blood leukocytes	*ALU* and *Satellite 2* repetitive elements, MethyLight-based assay	No association	• Prevalence stroke (baseline): no difference was observed in geometric mean methylation level between cases and controls (215 vs. 147, *p* = 0.15). • Incident stroke (follow-up): not analyzed.
(44)	Cohort: *n* = 712 (Stroke: Baseline: *n* = 51, Follow-up: *n* = 8)	Peripheral blood leukocytes	*LINE-1* repetitive elements, pyrosequencing	Hypomethylation	• Prevalent stroke (baseline): per 2.5% decrease in methylation level, OR = 1.90, 95% CI: 1.16–3.10. • Incident stroke (follow-up): per 2.5% decrease in methylation level, OR = 1.80; 95% CI, 0.72–4.46.

*LINE-1, long-interspersed nuclear element 1; OR, odds ratio; CI, confidence interval*.

* *Methylation pattern refers to the methylation alteration of the site(s) in stroke cases compared to non-stroke controls*.

a *Overlapping participant*.

**Table 2:** Candidate-gene methylation analysis for ischemic stroke.

**Study**	**Sample size**	**Tissue**	**Methylation sites, platform**	Methylation pattern^*^	**Main relevant findings**
(26)^b^	Case: *n* = 12 Control: *n* = 12	Whole peripheral blood	*TNF-α* promoter (19 CpG sites), *PON* promoter (22 CpG sites), Sequenom EpiTYPER	Promoter hypomethylation	• Lower methylation level of *TNF-α* promoter was positively associated with stroke (OR = 9.0, 95% CI, 1.4–57.1, *p* = 0.020). • Methylation level of *PON* promoter was found to be interacting with energy intake in stroke cases (*p* = 0.017).
(28)^a^	Case: *n* = 201 Control: *n* = 217	Whole peripheral blood	*ERα* promoter (14 CpG sites), pyrosequencing	Promoter hypomethylation	• Mean methylation level: site 13, case vs. control, 2.60 vs. 3.05%, *p* = 0.035; site 14, case vs. control, 6.95 vs. 7.87%, *p* = 0.026. • Stroke subtypes, LAA/CE: site 5, case vs. control, 3.55 vs. 4.34%; site 9, case vs. control, 2.40 vs. 2.93%; site 12, case vs. control, 3.60 vs. 4.40%; site 13, case vs. control, 2.48 vs. 3.05%; site 14, case vs. control, 6.68 vs. 7.87%. (All *p* < 0.05).
(29)^a^	Case: *n* = 298 Control: *n* = 258	Whole peripheral blood	*MMP-2* promoter (8 CpG sites), pyrosequencing	Promoter hypomethylation	• Mean methylation level: all 8 sites, case vs. control, 3.24 vs. 3.64%; site 1, case vs. control, 1.88 vs. 2.13%; site 5, case vs. control, 1.92 vs. 2.25%; site 7, case vs. control, 2.70 vs. 3.02%; site 8, case vs. control, 4.46 vs. 4.98%. (All *p* < 0.05). • Stroke subtypes, small-vessel stroke: men, case vs. control, 3.01 vs. 3.65%, *p* = 0.018; women, not stated.
(30)	Case: *n* = 26 Control: *n* = 26	Whole peripheral blood	*APOE* promoter (17 CpG sites), pyrosequencing	Promoter hypermethylation	• Higher methylation level of site 16 was positively associated with ACI (OR = 16.1, 95% CI, 1.2–225.8, *p* = 0.039).
(31)	Case: *n* = 446 Control: *n* = 159	Whole peripheral blood	*MTHFR* gene (2 CpG sites), pyrosequencing	Hypermethylation	• Higher methylation level of CpG A was positively associated with stroke (OR = 4.73, 95% CI, 2.56–8.75, *p* < 0.001).
(32)	Case: *n* = 78 Control: *n* = 86	Whole peripheral blood	*TP53* promoter, Sanger sequencing	Promoter hypermethylation	• Mean methylation level: case vs. control, 32.1 vs. 16.3%; *p* < 0.001.
(33)	Case: *n* = 23 Control: *n* = 32	Peripheral blood leukocytes	*MIR-223* promoter (9 CpG sites), methylation-specific PCR	Promoter hypomethylation	• Lower mean methylation levels of a total of 7 CpG sites (sites 2, 3, 4, 6, 7, 8, 9) as well as island 1 and 2 of MIR-223 promoter were found in ACI cases compared to controls. (all *p* < 0.05).
(34)	Case: *n* = 120 Control: *n* = 80	Whole peripheral blood	*TM* promoter, methylation-specific PCR	Promoter hypermethylation	• Mean methylation level: case vs. control, 74.2 vs. 47.5%, *p* < 0.01.
(38)	Case: *n* = 55 Control: *n* = 55	Peripheral bloodleukocytes	*ABCG1* gene, *APOE* gene, pyrosequencing	Hypermethylation	• Higher DNA methylation at the *cg02494239* site in *ABCG1* was positively associated with stroke (OR = 2.416, 95% CI: 1.024–5.700, *p* = 0.044). • No associations were observed for the *cg06500161* site in *ABCG1* and the *cg14123992* site in *APOE*.
(41)	Case: *n* = 132 Control: *n* = 218	Whole peripheral blood	*CBS* promoter, methylation-specific PCR	Promoter hypermethylation	• Median methylation level: case vs. control, 38.05 vs. 30.53%, *p* < 0.001. • Higher methylation level of *CBS* promoter was positively associated with stroke. (OR = 1.015 95% CI: 1.003–1.028).
(42)	Case: *n* = 64 Control: *n* = 138	Whole peripheral blood	*AHCY* gene, methylation-specific PCR	Hypermethylation	• Median methylation level: case vs. control, 0.13 vs. 0.06%, *p* < 0.0001.

*ABCG1, ATP-binding cassette G1; AHCY, S-adenosylhomocysteine hydrolase; APOE, Apolipoprotein E; CBS, Cystathionine β-synthase; ERα, estrogen receptor alpha; MIR-223, microRNA223; MMP-2, Matrix metalloproteinase-2; MTHFR, methylenetetrahydrofolate reductase; PON, paraoxonase; TM, thrombomodulin; TNF-α, human tumor necrosis factor; TP53, tumor protein p53; LAA/CE, large-artery atherosclerosis and cardioembolism; ACI, atherosclerotic cerebral infarction; OR, odds ratio; CI, confidence interval*.

* *Methylation pattern refers to the methylation alteration of the site(s) in stroke cases compared to non-stroke controls*.

a *Overlapping participant*;

b *Overlapping participant*.

**Table 3:** Epigenome-wide methylation analysis for ischemic stroke.

**Study**	**Sample size**	**Tissue**	**Methylation site, platform**	Methylation pattern^*^	**Main relevant findings**
(36)^c^	Case: *n* = 82 Control: *n* = 41	Whole peripheral blood	DNA methylation age: Horvath's method: 353 methylation probes; Hannum's method: 71 methylation probes, Illumina HM450K	Differentially methylated	• Hannum age acceleration was positively associated with stroke (OR = 1.13, 95%CI: 1.00–1.26, *p* = 0.045). • No association was observed between Horvath age acceleration and stroke.
(27)	Case: *n* = 161 Control: *n* = 161	Whole peripheral blood	485,578 CpG sites, Illumina HM450K, pyrosequencing and Sequenom EpiTYPER	Hypermethylation	• In the validation study, higher methylation levels of *HLA-DRB1* and *HLA-DQB1* were found in stroke cases compared to control (both *p* < 0.05).
(37)^b^	Case: *n* = 72 Control: *n* = 67	Peripheral blood leukocytes	27,578 CpG sites, Illumina HM27K	Hypermethylation	• 80 CpG sites were found to be differentially methylated in stroke cases compared to controls, and 56 CpG sites were found to be interacting with obesity in stroke cases. • In validation study, higher methylate on levels at CpG site 19_20 in *WT1*, at the CpG site 1_2_10, 11, 12, 13, 14, 16_17, 18, 22 in *PM20D1* promoter were found in stroke cases compared to controls (all *p* < 0.05). • In the validation study, CpG site 1 and 8 in *CALD1* and CpG site 8_9 in *KCNQ1* were found to be interacting with obesity in stroke cases. • In the subgroup analysis, higher methylation levels of CPG 1_2_3 was found to be correlated with NIHSS (*P* = 0.016, *r* = 0.371).
(40)^c^	Case: *n* = 548 Control: *n* = 245	Whole peripheral blood	358,709 CpG sites, Infinium MethylationEPIC Beadchip and Illumina HM450K	Hypermethylation	• In validation study, higher methylation levels of 22 CpGs in 21 loci (*CAMSAP3, SLC35E1, ZFHX3, PIM3, MAPK1, LRRC26, HIF1A, RNF126, SENP3, ANAPC11, PLBD2, CCNL2, PUM1, ITPKB, NAPA, IL15RA, ACSL1, JMY, PUF60, CHSY1*, and *BAMBI*) were found in stroke cases compared to controls (all *p* < 0.000).
(39)	Case: *n* = 301 Control: *n* = 313	Whole peripheral blood	482,360 sites CpG sites, Illumina HM450K	Differentially methylated	• In the validation study, lower methylation levels of 438 CpG loci were found in stroke cases (mean difference, −7%, 95%CI, −5 to −38.7%). • In the validation study, higher methylation levels of 574 CpG loci were found in stroke cases (mean methylation, 6.8%, 95%CI, 5 to 28.9%). • In the validation study, lower average methylation level of 7 CpG sites (including CpG_3, CpG_4.5, CpG_8, CpG_9, CpG_10, CpG_11, CpG_13.14, and CpG_16) in *MTRNR2L8* was found in LAA stroke cases (mean difference: −13.01%, *p* < 0.000).
(45)	Cohort: *n* = 729 (Stroke: *n* = 27)	Whole peripheral blood	470,789 CpG sites, Illumina HM450K	No association	• No association between CpG methylation level and stroke was reported.

*ACSL1, Long-chain-fatty-acid–CoA ligase 1; ANAPC11, Anaphase-promoting complex subunit 11; BAMBI, BMP and activin membrane-bound inhibitor homolog; CALD1, Caldesmon 1; CAMSAP3, Calmodulin-regulated spectrin-associated protein 3; CCNL2, Cyclin-L2; CHSY1, Chondroitin sulfate synthase 1; HLA-DRB1, human leukocyte antigen DR beta 1; HLA-DQB1, human leukocyte antigen DQ beta 1; HIF1A, Hypoxia-inducible factor 1-alpha; ITPKB, Inositol-trisphosphate 3-kinase B; IL15RA, Interleukin-15 receptor subunit alpha; JMY, Junction-mediating and -regulatory; LRRC26, Leucine-rich repeat-containing protein 26; KCNQ1, potassium voltage-gated channel, KQT-like subfamily,member 1; MAPK1, Mitogen-activated protein kinase 1; PIM3, Serine/threonine-protein kinase pim-3; PM20D1, peptidase M20 domain containing 1; PLBD2, Putative phospholipase B-like 2; PUM1, Pumilio homolog 1; PUF60, Poly(U)-binding-splicing factor PUF60; RNF126, RING finger protein 126; SENP3, Sentrin-specific protease 3; SLC35E1, Solute carrier family 35 member E1; WT1, wilms' tumor 1; ZFHX3, Zinc finger homeobox protein 3; HM450K, Infinium HumanMethylation450 BeadChip; HM27K, Infinium HumanMethylation27 BeadChip; NIHSS, the National Institute of Health Stroke Scale; OR, odds ratio; CI, confidence interval*.

* *Methylation pattern refers to the methylation alteration of the site(s) in stroke cases compared to non-stroke controls*.

b *Overlapping participant*;

c *Overlapping participant*.

### Global Methylation

A total of three studies measured global methylation level in relation to IS, with two using *LINE-1* repetitive elements and one using *ALU* plus *Satellite 2* repetitive elements. Among these three studies, one used a cross-sectional study design (35), and the other two used prospective study designs (43, 44) (Supplementary Table 2). However, one of prospective studies did not report the follow-up results of DNA methylation with IS (43). All three studies adjusted for risk factors to control the bias (Supplementary Table 2).

Findings of the association between global methylation and IS were summarized in Table 1. Two of the studies found that *LINE-1* was hypomethylated in IS patients. Specifically, Baccarelli et al. (44) indicated that lower *LINE-1* methylation level was associated with higher IS prevalence (every 2.5% 5-methylcytosine decrease in *LINE-1* methylation, OR = 1.90, 95%CI = 1.16–3.10), but this association did not reach statistical significance for IS incidence in follow-up analyses(every 2.5% 5-methylcytosine decrease in *LINE-1* methylation, OR = 1.80, 95%CI = 0.72–4.46). Lin et al. (35) also found that IS patients had lower *LINE-1* methylation level (IS vs. control: 75.9 vs. 77.0%, *p* = 0.0024). After the adjustment for risk factors, this association still existed in male patients (every 1% decrease in *LINE-1* methylation, OR = 1.20, 95%CI = 1.10–1.32). No associations were observed in the study estimating *ALU* and *Satellite 2* in relation to IS (43).

### Candidate Gene Methylation

The main findings from studies of candidate gene methylation were summarized in Table 2. There were 11 studies (case-control studies) that compared the methylation levels of candidate genes between the IS cases and controls. Eight studies measured DNA methylation in the promoter region and three measured in the gene body. Nine studies adjusted for risk factors to control the bias (Supplementary Table 2).

Overall, IS cases showed lower methylation levels in the promoter region of four genes, including human tumor necrosis factor (*TNF-*α) (26), estrogen receptor α (*ER*α) (28), matrix metalloproteinase-2 (*MMP-2*) (29), and microRNA223 (*MIR-223*) (33). In contrast, seven genes showed higher methylation levels in IS cases compared to controls. These include the promoter regions of apolipoprotein E (*APOE*) (30), tumor protein p53 (*TP53*) (32), thrombomodulin (*TM*) (34), and Cystathionine β-synthase (*CBS*) (41) and gene body of methylenetetrahydrofolate reductase (*MTHFR*) (31), ATP-binding cassette G1 (*ABCG1*) (38), and S-adenosylhomocysteine hydrolase (*AHCY*) (42). The methylation level of Paraoxonase (*PON*) promoter was observed to be interacting with energy intake in IS cases (26). No associations were observed in the gene body of *APOE* methylation and IS (38). Furthermore, two studies conducted sub-analyses according to stroke subtypes. One found that women with large-artery atherosclerosis and cardio-embolic (LAA/CE) had lower methylation levels of *ER*α promoter (28), and the other one found *MMP-2* promoter being hypomethylated in men with small-vessel stroke (29).

### Epigenome-Wide Methylation

The main findings of the epigenome-wide association studies (EWAS) were summarized in Table 3. Six studies (five case-control studies and one cross-sectional study) used this hypothesis-free approach. Specifically, one case-control study investigated IS in relation to two types of DNA methylation age, which were calculated according to the methylation level of 353 and 71 CpG probes across the genome, respectively (36). It found that individuals with an IS diagnosis had higher age acceleration (the residual results from regressing DNA methylation age on chronological age) in the epigenetic clock calculated by Hannum's method. However, no associations were observed when using Havorth's method. The other five studies identified potential DNA methylation sites without a pre-specified hypothesis that they were related to IS. Among which, a maximum of 438 CpG sites were identified to be differentially methylated in IS cases compared to controls. Four studies conducted validation tests, and 26 candidate genes were detected to be associated with IS (27, 37, 39, 40). However, one study observed no association between methylation and IS (45). Three studies conducted enrichment analyses showing that the identified loci were related to several biological pathways including, inflammation, angiogenesis, metabolic, and immune-related function (27, 39, 40).

## Discussion

This systematic review involving 7,014 individuals suggested that DNA methylation appeared to be associated with the occurrence of IS. Thirty-two genes were found to be hypermethylated and five were hypomethylated in stroke cases compared to controls. *LINE-1* methylation and epigenetic clock also showed an association with IS. Gender might influence the differences of DNA methylation levels in stroke subtypes, such as LAA/CE and small-vessel stroke. Energy taking and obesity were found to be interacting with the methylation level of specific loci in IS cases.

### Methods of DNA Methylation Analysis

According to different laboratory processes, conditions, and methods of data analysis, DNA methylation analysis can be categorized into three main types, including global, gene-specific, and epigenome-wide methylation analysis (46). All studies included in this review used one of the methods to measure the DNA methylation accordingly and explored their relationship with IS.

Global methylation refers to the overall level of 5-methylcytosine content in the genome. This is mostly measured using repetitive elements which constitute approximately 55% of the human genome and account for a significant fraction of DNA methylation in human (47–49). Analyses of candidate gene methylation, on the other hand, are focused on the methylation level of one or more specific sites which were pre-selected based on their possible involvement in the pathological mechanism (50). This analysis approach investigates the role of epigenetic modifications according to the functions of these candidate genes. In terms of epigenome-wide methylation, studies of this type utilize wide arrays to quantify the DNA methylation level of particular sites across the genome, in order to discover disease-associated methylated sites without a predilection on specific loci using a hypothesis-free approach. Due to such heterogeneity in analysis methodologies, it is difficult to directly compare the results of previous studies, especially for external validation, even though great efforts have been made to help standardize the analysis approach and improve the reliability of the findings (51).

### Global DNA Methylation

#### Global DNA Methylation and Stroke

Methylation levels of *LINE-1, ALU* and *Satellite 2* repeats were found to be significantly associated with global DNA methylation as measured by high-performance liquid chromatography (52). Therefore, the average methylation levels of these elements are commonly considered as surrogates to express the methylation level of total cytosine in the genome (53, 54). Two studies included in the present review found that individuals with IS had lower methylation level of LINE-1, and it is worth noting that one of them observed this association prospectively. The consistent trend of association found in the robust cohort study could provide stronger evidence that *LINE-1* hypomethylation plays an important role in the pathogenesis of IS at a pre-clinical stage in the asymptomatic individuals and thus might be considered as an early etiologic factor.

#### Possible Pathological Mechanisms of Global Methylation

*LINE-1* accounts for approximately 17% of the genome sequence, and it is the only type of elements that can be activated in *LINE* families (49). Hypomethylated alternation of *LINE-1* in somatic cells may trigger genomic instability and gene deregulation, which then alters gene coding and expression (55, 56). Although the biological function of *LINE-1* has not been fully understood, there have been a few studies suggesting that its hypomethylated alternation is associated with risk factors of stroke. According to previous studies, *LINE-1* hypomethylation could modify the metabolism of lipid and carbohydrate and cause aberrant lipid profile and impaired glucose metabolism, which might further lead to the formation of atherosclerotic plaques (55, 57–61). Additionally, *LINE-1* methylation was reported to become lower with age, which might reflect the cumulated effects of age-related environmental risk factors on the onset of stroke (55). The other study focusing on the methylation of *ALU* and *Satellite 2* repetitive elements, on the other hand, failed to observe an association. *ALU* and *Satellite 2* were also reported to be associated with risk factors of IS, including higher BMI and blood pressure in previous studies (54, 61). The non-significant association might be due to the underpowered analysis since there were only four stroke cases included in the analysis. On the other hand, a combined measurement of *ALU* and Satellite for global DNA methylation analysis differs from the measurement of *LINE-1* in the assay and genetic position. Previous studies indicated that the methylation levels of *ALU* and *LINE-1* were correlated only in cancer cells rather than peripheral blood cells, and their regulatory mechanisms regarding DNA methylation might be different (62, 63). Therefore, *ALU* and *Satellite 2* might show different traits and patterns in relation to cerebral ischemia.

Global DNA methylation of repetitive elements can be a novel marker for stroke as standardized assays of DNA methylation are available, and they can nicely reflect global DNA methylation changes. However, there are concerns to be considered. The methylation alternation of the promoter and gene body might have different effects on gene expression. For example, hypermethylation in the gene body normally increases gene expression, while higher methylation in CpG islands of a promoter mostly leads to lower gene expression (54). Global DNA methylation of repetitive elements could only provide a rough measurement of methylation patterns. Some correlations between methylation of repetitive elements and specific genes were found, but the majority of these studies focused on other diseases, such as gastritis and glioma (64–66). Therefore, further studies are encouraged to expand the mechanisms of global methylation to specific loci and investigate their inter-relationship.

### Genome-Wide and Candidate Gene Methylation

#### Genome-Wide and Gene-Specific DNA Methylation and Stroke

Genome-wide and candidate gene methylation studies identified a total of 37 genes that were differentially methylated between stroke cases and controls. Twenty-five genes identified in genome-wide association studies were validated in replication samples, while none of the findings in candidate-gene studies was validated by other studies.

#### Possible Pathological Mechanisms of Genome-Wide and Gene-Specific DNA Methylation

The influence of DNA methylation alternations on the pathogenesis of IS has been studied in the past few years. Generally, methylation alternations of these genes regulate gene expression and risk factors of IS via a variety of pathological processes, such as disorders of the coagulation cascade, higher plasma homocysteine, dyslipidemia, atherosclerosis, and inflammatory response (54). For example, *APOE* controls an essential enzyme for lipid profile, and it was found that *APOE* genotype, especially the E4 allele, was associated with a higher level of LDL-C and carotid intima-media thickness (67–69). The hypermethylation of the *APOE* promoter can cause aberrant expression of the *APOE* gene, eventually leading to dyslipidemia and earlier onset of stroke.

DNA methylation alternations might also be involved in the pathway of the inflammatory response and cell death. For example, *TNF-*α has detrimental effects on both neuronal and glial cells by disrupting the blood-brain barrier or activating cell death signaling pathways (70). The lower methylation level of *TNF-*α promoter might increase the expression of *TNF-*α in stroke patients, causing glutamate excitotoxicity and apoptosis on neurons (71). Likewise, the genes of *TP53, HLA-DRB1*, and *HLA-DQB1* were also found to be associated with early neurological deterioration in IS for its function of regulating cell proliferation in atherosclerotic plaques (72, 73). Aberrant methylation on these genes might lead to an inflammatory response in arteries and the formation of arterial plaques, which accelerates the formation of atherosclerosis.

DNA methylation might also elevate the level of plasma homocysteine, which is one of the most established risk factors for stroke (74). *CBS* is a major enzyme in the metabolism of homocysteine converting to cysteine, and its deficiency could cause hyperhomocysteinemia (74). Hypermethylated *CBS* promoter might silence *CBS* gene expression and subsequently reduce enzyme activity, leading to plasma homocysteine accumulation and increased risk of stroke (75). Another possible mechanism is related to disorders of coagulation cascade. *TM* gene acts as a cofactor of thrombin and reduces blood coagulation, and its deficiency might cause cerebral thrombosis for less inhibited coagulation and fibrinolysis (76).

However, the biological mechanism affected by gene expression might only partially explain the mechanisms of stroke onset. For example, *MTHFR* is a major enzyme in the metabolism of vitamin folate, and its deficiency caused by hypermethylation might lead to hyperhomocysteinemia and subsequently increase the risk of stroke (6, 77). Meanwhile, hypermethylated *MTHFR* gene might function as a mediator on a broader pathological pathway, synergistically leading to hypermethylation of the *TM* gene promoter and further inducing *TM* gene silencing (34). On the other hand, Wei et al. (31) failed to observe an association of *MTHFR* methylation with plasma homocysteine despite its association with IS in this study. Therefore, there are still some underlying mechanisms unexplored, suggesting that new biological pathways as well as new loci related to the risk factors and onset of IS are needed to be explored.

#### Epigenetic Clock and Stroke

One of the included studies found that age acceleration using Hannum's method was positively associated with stroke (36). As the epigenetic clock is an algorithm calculated by the methylation level at a number of age-related loci across the human genome (78), it works as a marker of biological age against chronological age. Acceleration in biological age is probably a better scale for aging than the chronological age, and it is associated with many diseases as well as mortality risk (79). Since aging is an extremely chronic process influenced complicatedly by a large number of environmental factors (80, 81), Hannum's epigenetic clock could be a marker for the interaction between brain aging and the environment in relation to stroke.

### Limitations of the Current Evidence

First, among the included studies in the present review, only one study used prospective data to identify the association of DNA methylation with IS while the other studies basically used case-control or cross-sectional study design. Such study designs could not provide robust evidence for the temporality or causality of associations presented as reverse causations might have existed. Although some of the pathological processes mentioned above could provide evidence for the biological function, these are not enough to demonstrate the causal relationship due to the complexity of the pathological mechanisms. Second, in terms of the collection of bio-samples, all studies collected DNA from the blood while it is unclear if DNA methylation in blood could accurately reflect its level in brain tissues. Moreover, given the dynamic nature of the epigenetic modification, the level of DNA methylation might change as the disease progresses. Therefore, the amount of time between the onset of stroke and collection of bio-sample is an important factor that reflects the potentially changeable role of DNA methylation in different phases before and after stroke. The majority of the included studies assessed DNA methylation at an acute/subacute stage of stroke. No study measured DNA methylation longitudinally or recorded the exact time interval between stroke and bio-sample collection, leaving an open question of how DNA methylation would change through the course of the disease. This could be an important consideration for future studies. Third, the homogeneity of the study sample might have limited the generalizability of the results. For example, 13 out of 20 studies used samples which are comprised exclusively of Asians. Also, several studies used the data from overlapping participants.

### Prospective

In order to improve the quality of evidence, it is preferable to investigate this research question by a large-scale prospective cohort study with comprehensive data collection in a well-defined healthy population. First, longitudinal observation of the trajectory of DNA methylation might help to reflect the environment-gene interaction, especially the cumulated environmental effects on DNA methylation over time. Also, such a study design with a baseline as well as multiple measurements of DNA methylation during follow-up in healthy individuals provides evidence for the longitudinal properties of DNA methylation and its complex interaction with cerebral ischemia. It delineates a more comprehensive epigenetic pathway for stroke prevention and recovery. For example, in addition to only focusing on the association between DNA methylation and occurrence of stroke, one study also found that methylation at the baseline was correlated with stroke severity at hospital admission, suggesting that it might be predictive of the functional loss/recovery after admission (37). Therefore, it is a promising direction for future studies to investigate how DNA methylation at the early stage of stroke is associated with the short- or long-term functional outcome of IS using a patient sample. Second, studies with large sample size and sufficient length of follow-up are important, as the prospective study included in this review only observed eight IS cases during follow-up, which might have caused underpowered analysis. Third, it is crucial to standardize the methods of DNA methylation measure for reliable comparison of results across different studies. Fourth, a large number of covariates have been adjusted in data analyses of the previous studies, which is done most likely to control for the confounding effects. For example, many risk factors of IS that were adjusted in the included studies, such as BMI/obesity, blood pressure/hypertension, and smoking are proved to be associated with both cerebral ischemia (82) and the level of methylation (60, 83–85). However, the covariates considered in previous studies have been highly heterogeneous, possibly due to data availability. Some studies collected the data of physical examination and history of disease from the study participants (31, 41, 44), while some only had basic information (32). For future studies, it is important to adequately collect and adjust a wide range of potential confounders, including demographic, lifestyle, and health data at both baseline and follow-up, to avoid residual confounding bias.

The clinical utility of epigenetic biomarker is promising as techniques in epigenome-sequencing is rapidly developing. Since DNA methylation is involved in many pathological pathways related to the well-established risk/protective factors of stroke, epigenetic biomarker has the potential to become a prediction tool to identify people at risk of stroke at the asymptomatic stage. Also, a better understanding of how DNA methylation interplays with metabolism, inflammation, or other pathways will help supplement the current treatment of IS. Epigenetic therapies that target the post-translational stage by modifying DNA methylation might be of huge clinical value for stroke patients, as aforementioned evidence has indicated genetic causes of cerebral ischemia and potential effects of DNA methylation. Some epigenetic therapies have already been used clinically for cancer patients (86). It is possible to prioritize and explore epigenetic therapies for ischemic stroke.

### Strengths and Limitations of this Review

To our knowledge, this study involving more than 7,000 participants, is the first study systematically and comprehensively summarizing the current evidence of the relationship of DNA methylation with IS. We identified the studies from four major databases as well as other sources manually and extracted all the relevant information from these studies. Also, we performed quality assessments for these studies using an established tool. However, two limitations need to be acknowledged. First, we only focus on ischemic stroke, which might cause the omission hemorrhagic stroke. Second, due to high heterogeneity, we were unable to perform meta-analyses and use Begg's funnel plot and Egger's test to examine publication bias.

## Conclusion

This review systematically integrated current evidence for the role of DNA methylation in IS. Epigenetic clock and methylation of *LINE-1* repetitive elements, as well as a number of genes, were found to be associated with IS. However, conclusive evidence has not yet been drawn due to high heterogeneity across studies, uncertain causal relationship, and complex process of the pathogenesis of IS. Future studies with a large-scale, prospective design, comprehensive data collection, and robust methylation measures, might help answer the research question.

## Data Availability Statement

All datasets generated for this study are included in the article/Supplementary Material.

## Author Contributions

MZ was involved in study conception and design, collection and analysis of data, and draft writing. JZ carried out study design, collection and analysis of data, and critical revision of the manuscript. XZ, HQ, XX, and JW performed study design and critical revision of the manuscript. ZL and JH were involved in study conception and design, collection and analysis of data, and critical revision of the manuscript. All authors contributed to the article and approved the submitted version.

## Conflict of Interest

The authors declare that the research was conducted in the absence of any commercial or financial relationships that could be construed as a potential conflict of interest.

## References

[1] GBD 2016 Neurology Collaborators Global, regional, and national burden of stroke, 1990-2016: a systematic analysis for the Global Burden of Disease Study 2016. Lancet Neurol. (2019) 18:439–58. 10.1016/s1474-4422(19)30034-1 30871944 PMC6494974

[2] Krishnamurthi RV Moran AE Feigin VL Barker-Collo S Norrving B Mensah GA Stroke prevalence, mortality and disability-adjusted life years in adults aged 20-64 years in 1990-2013: data from the global burden of disease 2013 study. Neuroepidemiology. (2015) 45:190–202. 10.1159/000441098 26505983

[3] Feigin VL Norrving B Mensah GA Global Burden of Stroke. Circ Res. (2017) 120:439–48. 10.1161/CIRCRESAHA.116.308413 28154096

[4] Singer J Gustafson D Cummings C Egelko A Mlabasati J Conigliaro A Independent ischemic stroke risk factors in older Americans: a systematic review. Aging (Albany NY). (2019) 11:3392–407. 10.18632/aging.101987 31127075 PMC6555455

[5] Dichgans M Pulit SL Rosand J Stroke genetics: discovery, biology, clinical applications. Lancet Neurol. (2019) 18:587–99. 10.1016/S1474-4422(19)30043-2 30975520

[6] Qureshi IA Mehler MF. Emerging role of epigenetics in stroke: part 1: DNA methylation and chromatin modifications. Arch Neurol. (2010) 67:1316–22. 10.1001/archneurol.2010.275 21060009 PMC3685873

[7] Feinberg A Epigenetics at the epicenter of modern medicine. JAMA. (2008) 299:1345–50. 10.1001/jama.299.11.1345 18349095

[8] Robertson KD DNA methylation and human disease. Nat Rev Genet. (2005) 6:597–610. 10.1038/nrg1655 16136652

[9] Martin EM Fry RC Environmental influences on the epigenome: exposure-associated DNA methylation in human populations. Annu Rev Public Health. (2018) 39:309–33. 10.1146/annurev-publhealth-040617-014629 29328878

[10] Alegria-Torres JA Baccarelli A Bollati V Epigenetics and lifestyle. Epigenomics. Jun. (2011) 3:267–77. 10.2217/epi.11.22 PMC3752894 22122337

[11] Lam K Pan K Linnekamp JF Medema JP Kandimalla R DNA methylation based biomarkers in colorectal cancer: a systematic review. Biochim Biophys Acta. (2016) 1866:106–20. 10.1016/j.bbcan.2016.07.001 27385266

[12] Jiang D Hong Q Shen Y Xu Y Zhu H Li Y The diagnostic value of DNA methylation in leukemia: a systematic review and meta-analysis. PLoS One. (2014) 9:e96822 10.1371/journal.pone.0096822 24810788 PMC4014555

[13] Guan Z Yu H Cuk K Zhang Y Brenner H Whole-blood DNA methylation markers in early detection of breast cancer: a systematic literature review. Cancer Epidemiol Biomarkers Prev. (2019) 28:496–505. 10.1158/1055-9965.EPI-18-0378 30487132

[14] Gurung PMS Barnett AR Wilson JS Hudson J Ward DG Messing EM Prognostic DNA methylation biomarkers in high-risk non-muscle-invasive bladder cancer: a systematic review to identify loci for prospective validation. Eur Urol Focus. (2020) 6:683–97. 10.1016/j.euf.2019.02.012 30803927

[15] Teroganova N Girshkin L Suter CM Green MJ DNA methylation in peripheral tissue of schizophrenia and bipolar disorder: a systematic review. BMC Genet. (2016) 17:27 10.1186/s12863-016-0332-2 26809779 PMC4727379

[16] Fransquet PD Lacaze P Saffery R McNeil J Woods R Ryan J Blood DNA methylation as a potential biomarker of dementia: a systematic review. Alzheimers Dement. (2018) 14:81–103. 10.1016/j.jalz.2017.10.002 29127806 PMC6810631

[17] Endres M Fan G Meisel A Dirnagl U Jaenisch R Effects of cerebral ischemia in mice lacking DNA methyltransferase 1 in post-mitotic neurons. Neuroreport. (2001) 12:3763–6. 10.1097/00001756-200112040-00032 11726790

[18] Gapp K Woldemichael BT Bohacek J Mansuy IM. Epigenetic regulation in neurodevelopment and neurodegenerative diseases. Neuroscience. (2014) 264:99–111. 10.1016/j.neuroscience.2012.11.040 23256926

[19] Casas JP Hingorani AD Bautista LE Sharma P. Meta-analysis of genetic studies in ischemic stroke: thirty-two genes involving approximately 18,000 cases and 58,000 controls. Arch Neurol. (2004) 61:1652–61. 10.1001/archneur.61.11.1652 15534175

[20] Ghosh J Coutifaris C Sapienza C Mainigi M Global DNA methylation levels are altered by modifiable clinical manipulations in assisted reproductive technologies. Clin Epigenet. (2017) 9:14 10.1186/s13148-017-0318-6 PMC5295214 28191261

[21] Lim U Song MA. Dietary and lifestyle factors of DNA methylation. Cancer Epigenet. (2012) 863:359–76. 10.1007/978-1-61779-612-8_23 22359306

[22] Moher D Shamseer L Clarke M Ghersi D Liberati A Petticrew M. Preferred reporting items for systematic review and meta-analysis protocols (PRISMA-P) 2015 statement. Syst Rev. (2015) 4:1. 10.1186/2046-4053-4-1 25554246 PMC4320440

[23] Pfalzer AC Choi SW Tammen SA Park LK Bottiglieri T Parnell LD. S-adenosylmethionine mediates inhibition of inflammatory response and changes in DNA methylation in human macrophages. Physiol Genomics. (2014) 46:617–23. 10.1152/physiolgenomics.00056.2014 25180283

[24] Feltus FA Lee EK Costello JF Plass C Vertino PM. Predicting aberrant CpG island methylation. Proc Natl Acad Sci U S A. (2002) 100:12253–8. 10.1073/pnas.2037852100 14519846 PMC218745

[25] Wells G Shea B O'Connell D The Newcastle-Ottawa Scale (NOS) for Assessing the Quality of Nonrandomised Studies in Meta-Analyses. (2015) Available online at: http://www.ohri.ca/programs/clinical_epidemiology/nos_manual.pdf.

[26] Gomez-Uriz AM Goyenechea E Campion J Arce A Martinez MT Puchau B Epigenetic patterns of two gene promoters (TNF-alpha and PON) in stroke considering obesity condition and dietary intake. J Physiol Biochem. (2014) 70:603–14. 10.1007/s13105-014-0316-5 24500802

[27] Deng GX Xu N Huang Q Tan JY Zhang Z Li XF Association between promoter DNA methylation and gene expression in the pathogenesis of ischemic stroke. Aging (Albany NY). (2019) 11:7663–77. 10.18632/aging.102278 31527307 PMC6781986

[28] Lin HF Hsi E Liao YC Chhor B Hung J Juo SHH. Demethylation of circulating estrogen receptor alpha gene in cerebral ischemic stroke. (2015) PLoS One. 10:e0139608. 10.1371/journal.pone.0139608 26422690 PMC4589317

[29] Lin HF Hsi E Huang LC Liao YC Juo SH Lin RT. Methylation in the matrix metalloproteinase-2 gene is associated with cerebral ischemic stroke. J Investig Med. (2017) 65:794–9. 10.1136/jim-2016-000277 28193703

[30] Zhang H Zhao X Wang C Du R Wang X Fu J. A Preliminary Study of the association between apolipoprotein E promoter methylation and atherosclerotic cerebral infarction. J Stroke Cerebrovasc Dis. (2019) 28:1056–61. 10.1016/j.jstrokecerebrovasdis.2018.12.027 30658954

[31] Wei LK Sutherland H Au A Camilleri E Haupt LM Gan HS. A potential epigenetic marker mediating serum folate and vitamin B12 levels contributes to the risk of ischemic stroke. Biomed Res Int. (2015) 2015:167976. 10.1155/2015/167976 25705649 PMC4331165

[32] Wei Y Sun Z Wang Y Xie Z Xu S Xu Y. Methylation in the TP53 promoter is associated with ischemic stroke. Mol Med Rep. (2019) 20:1404–10. 10.3892/mmr.2019.10348 31173230

[33] Li Z Yu F Zhou X Zeng S Zhan Q Yuan M. Promoter hypomethylation of microRNA223 gene is associated with atherosclerotic cerebral infarction. Atherosclerosis. (2017) 263:237–43. 10.1016/j.atherosclerosis.2017.06.924 28683362

[34] Yang Z Wang L Zhang W Wang X Zhou S. Plasma homocysteine involved in methylation and expression of thrombomodulin in cerebral infarction. Biochem Biophys Res Commun. (2016) 473:1218–22. 10.1016/j.bbrc.2016.04.042 27079234

[35] Lin RT Hsi E Lin HF Liao YC Wang YS Juo SH. LINE-1 methylation is associated with an increased risk of ischemic stroke in men. Curr Neurovasc Res. (2014) 11:4–9. 10.2174/1567202610666131202145530 24295503

[36] Soriano-Tarraga C Giralt-Steinhauer E Mola-Caminal M Vivanco-Hidalgo RM Ois A Rodríguez-Campello A Ischemic stroke patients are biologically older than their chronological age. Aging (Albany NY). (2016) 8:2655–66. 10.18632/aging.101028 27922817 PMC5191861

[37] Gomez-Uriz AM Milagro FI Mansego ML Cordero P Abete I Arce AD Obesity and ischemic stroke modulate the methylation levels of KCNQ1 in white blood cells. Hum Mol Genet. (2015) 24:1432–40. 10.1093/hmg/ddu559 25429063

[38] Qin X Li J Wu T Wu Y Tang X Gao P Overall and sex-specific associations between methylation of the ABCG1 and APOE genes and ischemic stroke or other atherosclerosis-related traits in a sibling study of Chinese population. Clin Epigenet. (2019) 11:189 10.1186/s13148-019-0784-0 PMC6902418 31823830

[39] Shen Y Peng C Bai Q Ding Y Yi X Du H Epigenome-wide association study indicates hypomethylation of MTRNR2L8 in large-artery atherosclerosis stroke. Stroke. (2019) 50:1330–8. 10.1161/STROKEAHA.118.023436 31084332

[40] Soriano-Tarraga C Lazcano U. Identification of 20 novel loci associated with ischaemic stroke. Epigenome-wide association study. Epigenetics. (2020) 6:1–0. 10.1101/2019.12.11.872945 32202197 PMC7518691

[41] Wang C Xu G Wen Q Peng X Chen H Zhang J. CBS promoter hypermethylation increases the risk of hypertension and stroke. Clinics (São Paulo). (2019) 74:e630. 10.6061/clinics/2019/e630 30916171 PMC6438132

[42] Zhao L Chen X Zhou S Lin Z Yu X Huang Y. DNA methylation of AHCY may increase the risk of ischemic stroke. Bosn J Basic Med Sci. (2020) 20:471–6. 10.17305/bjbms.2020.4535 32020847 PMC7664786

[43] Kim M Long TI Arakawa K Wang R Yu MC Laird PW. DNA methylation as a biomarker for cardiovascular disease risk. PLoS One. (2010) 5:e9692. 10.1371/journal.pone.0009692 20300621 PMC2837739

[44] Baccarelli A Wright R Bollati V Litonjua A Zanobetti A Tarantini L. Ischemic heart disease and stroke in relation to blood DNA methylation. Epidemiology. (2010) 21:819–28. 10.1097/EDE.0b013e3181f20457 20805753 PMC3690659

[45] Rask-Andersen M Martinsson D Ahsan M Enroth S Ek WE Gyllensten U. Epigenome-wide association study reveals differential DNA methylation in individuals with a history of myocardial infarction. Hum Mol Genet. (2016) 25:4739–48. 10.1093/hmg/ddw302 28172975

[46] Rakyan VK Down TA Balding DJ Beck S. Epigenome-wide association studies for common human diseases. Nat Rev Genet. (2011) 12:529–41. 10.1038/nrg3000 21747404 PMC3508712

[47] Hwu HR Roberts JW Davidson EH Britten RJ Insertion and/or deletion of many repeated DNA sequences in human and higher ape evolution. Proc Natl Acad Sci U S A. (1986) 83:3875–9. 10.1073/pnas.83.11.3875 3012536 PMC323627

[48] Gu Z Wang H Nekrutenko A Li WH Densities, length proportions and other distributional features of repetitive sequences in the human genome estimated from 430 megabases of genomic sequence. Gene. (2000) 259:81–8. 10.1016/S0378-1119(00)00434-0 11163965

[49] Consortium^*^ IHGS Initial sequencing and analysis of the human genome. Nature. (2001) 409:860–921. 10.1038/35057062 11237011

[50] Shabalin AA Aberg KA van den Oord EJCG Candidate gene methylation studies are at high risk of erroneous conclusions. Epigenomics. (2015) 7:13–5. 10.2217/epi.14.70 25687462 PMC5503464

[51] Michels KB Binder AM Dedeurwaerder S Epstein CB Greally JM Gut I. Recommendations for the design and analysis of epigenome-wide association studies. Nat Methods. (2013) 10:949–55. 10.1038/nmeth.2632 24076989

[52] Weisenberger DJ Campan M Long TI Kim M Woods C Fiala E Analysis of repetitive element DNA methylation by MethyLight. Nucleic Acids Res. (2005) 33:6823–36. 10.1093/nar/gki987 16326863 PMC1301596

[53] Vryer R Saffery R What's in a name? Context-dependent significance of global methylation measures in human health and disease. Clin Epigenet. (2017) 9:1–4. 10.1186/s13148-017-0311-0 PMC5270354 28149330

[54] Muka T Koromani F Portilla E O'Connor A Bramer WM Troup J. The role of epigenetic modifications in cardiovascular disease: A systematic review. Int J Cardiol. (2016) 212:174–83. 10.1016/j.ijcard.2016.03.062 27038728

[55] Li W Shuchuan L Zhendong S Rongchao C Xiuping B Xueqi L. LINE-1 hypomethylation is associated with the risk of coronary heart disease in Chinese population. Arq Bras Cardiol. (2014) 102:481–8. 10.5935/abc.20140054 24918913 PMC4051451

[56] Schulz WA Steinhoff C AR F. Methylation of endogenous human retroelements in health and disease. Curr Top Microbiol Immunol. (2006) 310:211–50. 10.1007/3-540-31181-5_11 16909913

[57] Martin-Nunez GM Rubio-Martin E Cabrera-Mulero R Rojo-Martínez G Olveira G Valdés S. Type 2 diabetes mellitus in relation to global LINE-1 DNA methylation in peripheral blood: a cohort study. Epigenetics. (2014) 9:1322–8. 10.4161/15592294.2014.969617 25437047 PMC4622014

[58] Perng W Mora-Plazas M Marin C Rozek LS Baylin A Villamor E. A prospective study of LINE-1DNA methylation and development of adiposity in school-age children. PLoS One. (2013) 8:e62587. 10.1371/journal.pone.0062587 23638120 PMC3640064

[59] Valérie T André T Yves D Pérusse L Bélisle A Marceau S LINE-1 methylation in visceral adipose tissue of severely obese individuals is associated with metabolic syndrome status and related phenotypes. Clinical Epigenet. (2012) 4:10 10.1186/1868-7083-4-10 PMC3464682 22748066

[60] Cash HL McGarvey ST Houseman EA Marsit CJ Hawley NL Lambert-Messerlian GM. Cardiovascular disease risk factors and DNA methylation at the LINE-1 repeat region in peripheral blood from Samoan Islanders. Epigenetics. (2011) 6:1257–64. 10.4161/epi.6.10.17728 21937883 PMC3225843

[61] Alexeeff SE Baccarelli AA Halonen J Coull BA Wright RO Tarantini L Association between blood pressure and DNA methylation of retrotransposons and pro-inflammatory genes. Int J Epidemiol. (2013) 42:270–80. 10.1093/ije/dys220 23508416 PMC3600626

[62] Hou L Wang H Sartori S Gawron A Lissowska J Bollati V. Blood leukocyte DNA hypomethylation and gastric cancer risk in a high-risk Polish population. Int J cancer. (2010) 127:1866–74. 10.1002/ijc.25190 20099281 PMC3009461

[63] Choi JY James SR Link PA McCann SE Hong CC Warren D Association between global DNA hypomethylation in leukocytes and risk of breast cancer. Carcinogenesis. (2009) 30:1889–97. 10.1093/carcin/bgp143 19584139 PMC2783000

[64] Yamamoto E Toyota M Suzuki H Kondo Y Sanomura T Murayama Y LINE-1 hypomethylation is associated with increased CpG island methylation in Helicobacter pylori-related enlarged-fold gastritis. Cancer Epidemiol Biomarkers Prev. (2008) 17:2555–64. 10.1158/1055-9965.EPI-08-0112 18842996

[65] Martin-Nunez GM Cabrera-Mulero R Rubio-Martin E Rojo-Martinez G Olveira G Valdes S. Methylation levels of the SCD1 gene promoter and LINE-1 repeat region are associated with weight change: an intervention study. Mol Nutr Food Res. (2014) 58:1528–36. 10.1002/mnfr.201400079 24827925

[66] Fumiharu O Atsushi N Kazuya M Yugo K Yutaka K Tatsuya A The global DNA methylation surrogate LINE-1 methylation is correlated with MGMT promoter methylation and is a better prognostic factor for glioma. PLoS One. (2011) 6:e23332 10.1371/journal.pone.0023332 21829728 PMC3150434

[67] Lagging C Lorentzen E Stanne TM Pedersen A Soderholm M Cole JW APOE epsilon4 is associated with younger age at ischemic stroke onset but not with stroke outcome. Neurology. (2019) 93:849–53. 10.1212/WNL.0000000000008459 31619479 PMC6946482

[68] Khan TA Shah T Prieto D Zhang W Price J Fowkes GR. Apolipoprotein E genotype, cardiovascular biomarkers and risk of stroke: systematic review and meta-analysis of 14,015 stroke cases and pooled analysis of primary biomarker data from up to 60,883 individuals. Int J Epidemiol. (2013) 42:475–92. 10.1093/ije/dyt034 23569189 PMC3619955

[69] Satizabal CL Samieri C Davis-Plourde KL Voetsch B Aparicio HJ Pase MP. APOE and the Association of Fatty Acids With the Risk of Stroke, Coronary Heart Disease, and Mortality. Stroke. (2018) 49:2822–9. 10.1161/STROKEAHA.118.022132 30571417 PMC6310220

[70] Watters O O'Connor JJ. A role for tumor necrosis factor-alpha in ischemia and ischemic preconditioning. J Neuroinflammation. (2011) 8:87. 10.1186/1742-2094-8-87 21810263 PMC3161872

[71] Jones P. Functions of DNA methylation: islands, start sites, gene bodies and beyond. Nat Rev Genet. (2012) 13:484–92. 10.1038/nrg3230 22641018

[72] Gomez-Sanchez JC Delgado-Esteban M Rodriguez-Hernandez I Sobrino T Ossa NP Reverte S. The human Tp53 Arg72Pro polymorphism explains different functional prognosis in stroke. J Exp Med. (2011) 208:429–37. 10.1084/jem.20101523 21357744 PMC3058581

[73] Li J Chen G Gao X Shen C Zhou P Wu X. p53 participates in the protective effects of ischemic post-conditioning against OGD-reperfusion injury in primary cultured spinal cord neurous. Neurosci Lett. (2017) 638:129–34. 10.1016/j.neulet.2016.12.037 27993707

[74] Stanzione R Cotugno M Bianchi F Marchitti S Forte M Volpe M Pathogenesis of ischemic stroke: role of epigenetic mechanisms. Genes. (2020) 11:89 10.3390/genes11010089 PMC7017187 31941075

[75] Ding R Lin S Chen D. The association of cystathionine beta synthase (CBS) T833C polymorphism and the risk of stroke: a meta-analysis. J Neurol Sci. (2012) 312:26–30. 10.1016/j.jns.2011.08.029 21917271

[76] Boffa MC Karmochkine M. Thrombomodulin: an overview and potential implications in vascular disorders. Lupus. (1998) 7(Suppl. 2):S120–5. 10.1177/096120339800700227 9814688

[77] Parnetti L Caso V Santucci A Corea F Lanari A Floridi A. Mild hyperhomocysteinemia is a risk-factor in all etiological subtypes of stroke. Neurol Sci. (2004) 25:13–7. 10.1007/s10072-004-0219-5 15060810

[78] Armstrong NJ Mather KA TA Wright MJ Trollor JN Ames D Brodaty H. Aging, exceptional longevity and comparisons of the Hannum and Horvath epigenetic clocks. Epigenomics. (2017) 9:689–700. 10.2217/epi-2016-0179 28470125

[79] Fransquet PD Wrigglesworth J Woods RL Ernst ME Ryan J. The epigenetic clock as a predictor of disease and mortality risk: a systematic review and meta-analysis. Clin Epigenetics. (2019) 11:62. 10.1186/s13148-019-0656-7 30975202 PMC6458841

[80] Blagosklonny M. Answering the ultimate question What is the Proximal Cause of Aging? *Aging (Albany NY)*. (2012) 4:861–77. 10.18632/aging.100525 23425777 PMC3615154

[81] Fraga MF Esteller M. Epigenetics and aging: the targets and the marks. Trends Genet. (2007) 23:413–8. 10.1016/j.tig.2007.05.008 17559965

[82] Boehme AK Esenwa C Elkind MSV. Stroke risk factors, genetics, and prevention. Circ Res. (2017) 120:472–95. 10.1161/CIRCRESAHA.116.308398 28154098 PMC5321635

[83] Wang X Falkner B Zhu H Shi H Su S Xu X. A genome-wide methylation study on essential hypertension in young african american males. PLoS One. (2013) 8:e53938. 10.1371/journal.pone.0053938 23325143 PMC3542324

[84] Hironobu S Yoshifumi B Masayuki W Shiro I Keisuke M Takatsugu I LINE-1 hypomethylation in noncancerous esophageal mucosae is associated with smoking history. Ann Surg Oncol. (2012) 19:4238–43. 10.1245/s10434-012-2488-y 22766991

[85] Pearce MS McConnell JC Potter C Barrett LM Parker L Mathers JC. Global LINE-1 DNA methylation is associated with blood glycaemic and lipid profiles. Int J Epidemiol. (2012) 41:210–7. 10.1093/ije/dys020 22422454 PMC3304536

[86] Karimi M Johansson S Ekstrom TJ. Using LUMA: a Luminometric-based assay for global DNA-methylation. Epigenetics. (2006) 1:45–8. 10.4161/epi.1.1.2587 17998810

