# Supplementary material for: The Role of DNA Methylation in Ischemic Stroke: A Systematic Review
Source: Front Neurol. 2020 Oct 27;11:566124. doi: 10.3389/fneur.2020.566124 (PMC7652818; doi:10.3389/fneur.2020.566124)
Supplement: Supplementary file 1 [file Data_Sheet_1.docx]

**Supplementary**

| Supplementary Table 1. Search strategies | | |
| --- | --- | --- |
|  | **Keywords** | **Result** |
| EMBASE |  |  |
| 1 | Epigenomics/ | 62082 |
| 2 | DNA methylation/ | 80605 |
| 3 | CpG Islands/ | 23785 |
| 4 | S-Adenosylmethionine/ | 9824 |
| 5 | epigenetic*.mp. | 133386 |
| 6 | cpg.mp. | 51647 |
| 7 | s adenosylmethionine.mp. | 12871 |
| 8 | 1 or 2 or 3 or 4 or 5 or 6 or 7 | 208095 |
| 9 | stroke/ | 138525 |
| 10 | exp brain ischemia/ | 185801 |
| 11 | (cerebrovascular* adj3 accident*).mp. [mp=title, abstract, heading word, drug trade name, original title, device manufacturer, drug manufacturer, device trade name, keyword, floating subheading word, candidate term word] | 210215 |
| 12 | stroke*.mp. | 453503 |
| 13 | ((brain or cerebral) adj3 (ischemi* or ischaemi*)).mp. [mp=title, abstract, heading word, drug trade name, original title, device manufacturer, drug manufacturer, device trade name, keyword, floating subheading word, candidate term word] | 164393 |
| 14 | 9 or 10 or 11 or 12 or 13 | 595753 |
| 15 | 8 and 14 | 1354 |
| PUBMED | | |
| 1 | Search ((Epigenomics[mh] OR DNA methylation[mh] OR S-Adenosylmethionine[mh] OR CpG Islands[mh] OR s adenosylmethionine OR cpg OR epigenetic*[tiab]))) | 134249 |
| 2 | Search (stroke[mh] OR brain ischemia[mh] OR (cerebrovascular*[tiab] AND accident*[tiab]) OR stroke*[tiab] OR ((brain OR cerebral) AND (ischemi*[tiab] OR ischaemi*[tiab]))) | 354464 |
| 3 | Search (((((Epigenomics[mh] OR DNA methylation[mh] OR S-Adenosylmethionine[mh] OR CpG Islands[mh] OR s adenosylmethionine OR cpg OR epigenetic*[tiab]))))) AND (((stroke[mh] OR brain ischemia[mh] OR (cerebrovascular*[tiab] AND accident*[tiab]) OR stroke*[tiab] OR ((brain OR cerebral) AND (ischemi*[tiab] OR ischaemi*[tiab]))))) | 585 |

| Supplementary Table 1. Search strategies (continues) | | |
| --- | --- | --- |
|  | **Keywords** | **Result** |
| MEDLINE |  |  |
| 1 | Epigenomics/ | 5378 |
| 2 | DNA methylation/ | 47901 |
| 3 | CpG Islands/ | 12828 |
| 4 | S-Adenosylmethionine/ | 5441 |
| 5 | epigenetic*.mp. | 67205 |
| 6 | cpg.mp. | 30380 |
| 7 | s adenosylmethionine.mp. | 8693 |
| 8 | 1 or 2 or 3 or 4 or 5 or 6 or 7 | 116491 |
| 9 | stroke/ | 99537 |
| 10 | exp brain ischemia/ | 107513 |
| 11 | (cerebrovascular* adj3 accident*).mp. [mp=title, abstract, original title, name of substance word, subject heading word, floating sub-heading word, keyword heading word, organism supplementary concept word, protocol supplementary concept word, rare disease supplementary concept word, unique identifier, synonyms] | 6360 |
| 12 | stroke*.mp. | 258444 |
| 13 | ((brain or cerebral) adj3 (ischemi* or ischaemi*)).mp. [mp=title, abstract, original title, name of substance word, subject heading word, floating sub-heading word, keyword heading word, organism supplementary concept word, protocol supplementary concept word, rare disease supplementary concept word, unique identifier, synonyms] | 77685 |
| 14 | 9 or 10 or 11 or 12 or 13 | 330547 |
| 15 | 8 and 14 | 431 |

| Supplementary Table 1. Search strategies (continues) | | |
| --- | --- | --- |
|  | **Keywords** | **Result** |
| Cochrane Library | | |
| 1 | Epigenomics/ | 12 |
| 2 | DNA methylation/ | 233 |
| 3 | CpG Islands/ | 42 |
| 4 | S-Adenosylmethionine/ | 122 |
| 5 | epigenetic*.mp. | 1138 |
| 6 | cpg.mp. | 629 |
| 7 | s adenosylmethionine.mp. | 265 |
| 8 | 1 or 2 or 3 or 4 or 5 or 6 or 7 | 1977 |
| 9 | stroke/ | 8433 |
| 10 | exp brain ischemia/ | 3451 |
| 11 | (cerebrovascular* adj3 accident*).mp. [mp=title, original title, abstract, mesh headings, heading words, keyword] | 14044 |
| 12 | stroke*.mp. | 56017 |
| 13 | ((brain or cerebral) adj3 (ischemi* or ischaemi*)).mp. [mp=title, original title, abstract, mesh headings, heading words, keyword] | 7963 |
| 14 | 9 or 10 or 11 or 12 or 13 | 61470 |
| 15 | 8 and 14 | 28 |

| Supplementary Table 2. Characteristics of the included studies | | | | | | |
| --- | --- | --- | --- | --- | --- | --- |
| **Study** | **Study design** | **Place of recruitment** | **Mean age (SD), gender** | **Diagnosis approach** | **Stage, time** | **Adjustment for risk factors** |
| **Global methylation and risk of stroke** | | | | | | |
| (35) | Case-control  (Hospital-based) | Taiwan | Case: 61.1 (10.1) years  Control: 61.0 (10.9) years  Case: male (51.4%)  Control: male (48.2%) | Independent validation:  Laboratory examination + brain CT or MRI | Acute stroke, overnight | Adjustment for covariates in multivariable analysis in men: age, hypertension, diabetes, hyperlipidemia, and smoking |
| (43) | Prospective cohort  (Community-based) | Singapore | Age, NA  Case: male (50.0%)  Control: male (45.4%) | Self-report | Acute stroke, na | Age and gender |
| (44) | Prospective cohort  (Community-based) | America | Age, NA  Case: male (100%)  Control: male (100%) | Independent validation:  Medical records + Neurological evaluation + Cardiologist’s validation+ brain CT or MRI | Acute stroke, na | Age, body mass index, smoking history, alcohol drinking, diagnosis of hypertension, diastolic blood pressure, systolic blood pressure, diabetes, serum HDL, serum total cholesterol, statin use, plasma homocysteine, glomerular filtration rate, percent neutrophils, and lymphocytes in the differential white blood count |
| **Candidate-gene methylation and risk of stroke** | | | | | | |
| (26) | Case-control  (Hospital-based) | Spain | Case: 71.8 (11.7) years  Control: 72.4 (11.8) years  Case: male (50.0%)  Control: male (50.0%) | Independent validation: Neurological evaluation + brain CT or MRI | Acute stroke, na | Age, gender, and body mass index |
| (28) | Case-control  (Hospital-based) | Taiwan | Case: 60.8 (10.3) years  Control: 61.4 (11.0) years  Case: male (50.2%)  Control: male (46.5%) | Independent validation:  Laboratory examination + brain CT or MRI | Subacute stroke, na | Age, gender, smoking, hypertension, diabetes, and hypercholesterolemia |
|  | | | | | | |

| Supplementary Table 2. Characteristics of the included studies (continues) | | | | | | |
| --- | --- | --- | --- | --- | --- | --- |
| **Study** | **Study design** | **Recruitment place** | **Mean age (SD), gender** | **Diagnosis approach** | **Stage, time** | **Adjustment for risk factors** |
| **Candidate-gene methylation and risk of stroke** | | | | | | |
| (29) | Case-control  (Hospital-based) | Taiwan | Case: 61.5 (10.3) years  Control: 61.6 (10.5) years  Case: male (49.3%)  Control: male (46.9%) | Independent validation:  Laboratory examination + brain CT or MRI | Subacute stroke, na | Age, gender, smoking, hypertension, diabetes, and hypercholesterolemia |
| (30) | Case-control  (Hospital-based) | China | Case: 56.4 (10.4) years  Control:53.9 (11.0) years  Case: male (57.7%)  Control: male (50.0%) | Independent validation:  Neurological evaluation + brain MRI | Acute stroke, overnight | Age, gender, carotid atherosclerotic plaque, hypertension, HDL-C, homocysteine, and folate |
| (31) | Case-control  (Hospital-based) | Malaysia | Case: 52.1-52.6 years  Control: 51.1-52.7 years  Case: male (63.0%)  Control: male (52.2%) | Independent validation:  Brain CT or MRI | Acute stroke, na | Age, genders, waist to stature, diabetes, hypertension, hypercholesterolemia, heart diseases, smoking, coffee drinking, tea drinking, and serum vitamin profiles |
| (32) | Case-control  (Hospital-based) | China | Case: 61.5 (9.9) years  Control: 62.1 (7.1) years  Case: male (51.3%)  Control: male (57.0%) | Independent validation:  Magnetic resonance diffusion weighted imaging | Acute stroke, within 48h following stroke | Age |
| (33) | Case-control  (Hospital-based) | China | Case: 62.6 (11.5) years  Control: 57.0 (11.5) years  Case: male (74.0%)  Control: male (43.8%) | Independent validation:  Neurological evaluation + laboratory examination + brain MRI + angiography | Acute stroke, na | Systolic blood pressure, diastolic blood pressure, and total cholesterol |
| (34) | Case-control  (Hospital-based) | China | Case: 61.8 (12.0) years  Control: 61.3 (10.7) years  Case: male (64.2%)  Control: male (62.5%) | Independent validation:  Neurological evaluation + brain CT or MRI | Acute stroke, na | NA |
|  | | | | | | |

| Supplementary Table 2. Characteristics of the included studies (continues) | | | | | | |
| --- | --- | --- | --- | --- | --- | --- |
| **Study** | **Study design** | **Place of recruitment** | **Mean age (SD), gender** | **Diagnosis approach** | **Stage, time** | **Adjustment for risk factors** |
| **Candidate-gene methylation and risk of stroke** | | | | | | |
| (38) | Case-control  (family-based) | China | Case: 60.1 (7.6) years  Control: 59.7 (8.0) years  Case: male (67.3%)  Control: male (50.5%) | Independent validation:  Neurological evaluation + Brain CT or MRI | Acute stroke, na | gender, previous history of diabetes and hypertension, smoking, drinking, body mass index, and blood lipid levels |
| (41) | Case-control  (Hospital-based) | China | Case: 65.8 (9.3) years  Control: 64.4 (9.2) years  Case: male (59.1%)  Control: male (63.3%) | Independent validation: Neurological evaluation + brain CT or MRI | Acute stroke, na | Age, sex, body mass index, Hcy, uric acid, total cholesterol, triglycerides, glucose, waist circumference, hip circumference, systolic blood pressure, diastolic blood pressure, drinking history and smoking history |
| (42) | Case-control  (Hospital-based) | China | Case: 62.0 (11.5) years  Control: 63.3 (10.3) years  Case: male (54.7%)  Control: male (58.7%) | Independent validation:  Neurological evaluation + Brain CT or MRI | Acute stroke, overnight | NA |
| **Epigenome-wide association studies for risk of stroke** | | | | | | |
| (36) | Case-control  (Hospital-based) | Spain | Case: 63.9 (10.3) years  Control: 62.8 (14.2) years  Case: male (54.9%)  Control: male (51.2%) | Independent validation:  Brain CT or MRI | Acute stroke, na | Gender, diabetes mellitus, hypertension, atrial fibrillation, smoking habit and blood cell count associated to Hannum (NK, monocytes,  CD4+ T cells, naïve CD8 T cells and CD8+CD28‐CD45RA‐), and Horvath (NK, monocytes, CD8+ T cells, naïve CD8 and  CD4 T) |
| (27) | Case-control  (Hospital-based) | China | Case: 58.9 (9.2) years  Control: 58.2 (9.5) years  Case: male (31.7%)  Control: male (30.4%) | Independent validation:  Neurological evaluation + brain MRI | Acute stroke, na | NA |
|  | | | | | | |

| Supplementary Table 2. Characteristics of the included studies (continues) | | | | | | |
| --- | --- | --- | --- | --- | --- | --- |
| **Study** | **Study design** | **Place of recruitment** | **Mean age (SD), gender** | **Diagnosis approach** | **Stage, time** | **Adjustment for risk factors** |
| **Epigenome-wide association studies for risk of stroke** | | | | | | |
| (37) | Case-control  (Hospital-based) | Spain | Case:66.4-74.6 years  Control: 60.4-69.8 years  Case: male (47.2%)  Control: male (41.8%) | Independent validation: Neurological evaluation + brain CT or MRI | Acute stroke, na | Age, gender, and body mass index |
| (40) | Case-control  (Hospital-based) | Spain | Case: 71.0-75.0 years  Control:62.8-75.0 years  Case: male (61.9%)  Control: male (49.8%) | Independent validation: Neurological evaluation + brain CT or MRI | Acute stroke, na | Gender, diabetes mellitus, hypertension, atrial fibrillation, smoking habit |
| (39) | Case-control  (Hospital-based) | China | Case: 70.2-72.7 years  Control: 71.4-72.1 years  Case: male (47.8%)  Control: male (47.0%) | Independent validation: Neurological evaluation + brain CT or MRI | Acute stroke, within 72h following stroke | Age, sex, diabetes mellitus status, and hypertension |
| (45) | Cross-sectional (Community-based) | Sweden | 47.4 (NA) years  Male (46.8%) | Self-report | Chronic stroke, na | Age, gender, relatedness among individuals, estimated cell fractions, as well as array and slide information, and population stratification. |
| Abbreviation: NA/na, not available.  Note: the column of stage and time refers to the stage of ischemic stroke when bio-sample was collected and the time between disease onset and bio-sample collection. | | | | | | |

| Supplementary Table 3. Quality assessment of case-control studies by the Newcastle-Ottawa scale | | | | | | | | | | | | | | | | | |
| --- | --- | --- | --- | --- | --- | --- | --- | --- | --- | --- | --- | --- | --- | --- | --- | --- | --- |
| **Criteria**  **(case-control studies)** | **(35)** | **(26)** | **(28)** | **(29)** | **(30)** | **(31)** | **(32)** | **(33)** | **(34)** | **(38)** | **(41)** | **(42)** | **(36)** | **(40)** | **(27)** | **(37)** | **(39)** |
| 1) Is the case definition adequate? (independently validation scored 1 star) | 1 | 1 | 1 | 1 | 1 | 1 | 1 | 1 | 1 | 1 | 1 | 1 | 1 | 1 | 1 | 1 | 1 |
| 2) Representativeness of the cases (all subjects or random sampling scored 1 star) | 1 | 1 | 1 | 1 | 1 | 1 | 1 | 1 | 1 | 1 | 1 | 1 | 1 | 1 | 1 | 1 | 1 |
| 3) Selection of controls (community-based scored 1 star) | 1 | 0 | 1 | 1 | 0 | 0 | 0 | 0 | 0 | 0 | 0 | 0 | 0 | 0 | 0 | 0 | 0 |
| 4) Definition of controls (no history endpoint scored 1 star) | 1 | 1 | 1 | 1 | 1 | 1 | 1 | 1 | 1 | 1 | 1 | 1 | 1 | 1 | 1 | 1 | 1 |
| 5) Control for risk factors (control for important factor scored 1 star; control for additional factor scored 1 star) ^a^ | 2 | 2 | 2 | 2 | 2 | 2 | 1 | 2 | 0 | 2 | 2 | 0 | 2 | 2 | 0 | 2 | 2 |
| 6) Ascertainment of exposure (control for genotype and cell-type composition scored 1 star) | 1 | 1 | 1 | 1 | 0 | 0 | 1 | 1 | 1 | 1 | 1 | 1 | 1 | 1 | 1 | 1 | 1 |
| 7) Same method of ascertainment for cases and controls (scored 1 star) | 1 | 1 | 1 | 1 | 1 | 1 | 1 | 1 | 1 | 1 | 1 | 1 | 1 | 1 | 1 | 1 | 1 |
| 8) Non-response rate (same rate for both groups scored 1 star) | 1 | 1 | 1 | 1 | 1 | 1 | 1 | 1 | 1 | 1 | 1 | 1 | 1 | 1 | 1 | 1 | 1 |
| Total stars (maximum: 9) | 9 | 8 | 9 | 9 | 7 | 7 | 7 | 8 | 6 | 8 | 8 | 6 | 8 | 8 | 6 | 8 | 8 |
| Footnote: ^a^ A maximum of two points can be given for this item. | | | | | | | | | | | | | | | | | |

| Supplementary Table 4. Quality assessment of cohort studies and cross-sectional studies by the Newcastle-Ottawa scale | | | | |
| --- | --- | --- | --- | --- |
| **Criteria**  **(cohort studies)** | **(44)** | **Criteria**  **(cross-sectional studies)** | **(43)** ^b^ | **(45)** |
| 1) Representativeness of the exposed cohort (all subjects or random sampling scored 1 star) | 1 | 1) Representativeness of the sample (all subjects or random sampling scored 1 star) | 1 | 1 |
| 2) Selection of the non-exposed cohort (same community as the exposed cohort scored 1 star) | 1 | 2) Sample size (Justified and satisfactory scored 1 star) | 1 | 1 |
| 3) Ascertainment of exposure (control for genotype and cell-type composition scored 1 star) | 1 | 3) Non-respondents (response rate is satisfactory scored 1 star) | 1 | 1 |
| 4) Demonstration that outcome of interest was not present at start of study (scored 1 star) | 1 | 4) Ascertainment of the exposure (control for genotype scored 1 star and cell-type composition scored 1 star) ^a^ | 2 | 2 |
| 5) Control for risk factors (control for important factor scored 1 star; control for additional factor scored 1 star) ^a^ | 2 | 5) Control for risk factors (control for important factor scored 1 star; control for additional factor scored 1 star) ^a^ | 2 | 2 |
| 6) Assessment of outcome (independently validation scored 1 star) | 0 | 6) Assessment of the outcome (independently validation scored 1 star) | 1 | 0 |
| 7) Follow-up long enough for outcomes to occur (scored 1 star) | 1 | 7) Statistical test (data analysis including confidence intervals or p-value scored 1 star) | 1 | 1 |
| 8) Adequacy of follow up of cohorts (Complete follow up- all subject or lost to follow up unlikely to introduce bias scored 1 star) | 0 |  |  |  |
| Total stars (maximum: 9) | 7 | Total score (maximum: 9) | 9 | 8 |
| Footnote: ^a^ A maximum of two points can be given for this item. ^b^ The authors of M Kim (2010) did not report the follow-up results of DNA methylation with stroke. Thus this study was assessed by criteria for cross-sectional studies, although its study design is prospective. | | | | |
